# Influence of pharmacogenetics on response and toxicity in breast cancer patients treated with doxorubicin and cyclophosphamide

**DOI:** 10.1038/sj.bjc.6605587

**Published:** 2010-02-23

**Authors:** J Bray, J Sludden, M J Griffin, M Cole, M Verrill, D Jamieson, A V Boddy

**Affiliations:** 1 Northern Institute for Cancer Research, Paul O'Gorman Building, Medical School, Newcastle University, Newcastle upon Tyne, NE2 4HH, UK; 2 Northern Centre for Cancer Care, Newcastle Freeman Hospital, Newcastle upon Tyne, NE7 7DN, UK

**Keywords:** breast cancer, doxorubicin, cyclophosphamide, pharmacogenetics

## Abstract

**Background:**

Doxorubicin and cyclophosphamide (AC) therapy is an effective treatment for early-stage breast cancer. Doxorubicin is a substrate for ABCB1 and SLC22A16 transporters. Cyclophosphamide is a prodrug that requires oxidation to 4-hydroxycyclophosphamide, which yields a cytotoxic alkylating agent. The initial oxidation is catalysed by cytochrome P450 enzymes including CYP2B6, CYP2C9, CYP2C19 and CYP3A5. Polymorphic variants of the genes coding for these enzymes and transporters have been identified, which may influence the systemic pharmacology of the two drugs. It is not known whether this genetic variation has an impact on the efficacy or toxicity of AC therapy.

**Methods:**

Germ line DNA samples from 230 patients with breast cancer on AC therapy were genotyped for the following SNPs: ABCB1 C1236T, G2677T/A and C3435T, SLC22A16 A146G, T312C, T755C and T1226C, CYP2B6^*^2, ^*^8, ^*^9, ^*^3, ^*^4 and ^*^5, CYP2C9^*^2 and ^*^3, CYP3A5^*^3 and CYP2C19^*^2. Clinical data on survival, toxicity, demographics and pathology were collated.

**Results:**

A lower incidence of dose delay, indicative of less toxicity, was seen in carriers of the SLC22A16 A146G, T312C, T755C variants. In contrast, a higher incidence of dose delay was seen in carriers of the SLC22A16 1226C, CYP2B6^*^2 and CYP2B6^*^5 alleles. The ABCB1 2677A, CYP2B6^*^2, CYP 2B6^*^8, CYP 2B6^*^9, CYP 2B6^*^4 alleles were associated with a worse outcome.

**Conclusion:**

Variant alleles in the ABCB1, SLC22A16 and CYP2B6 genes are associated with response to AC therapy in the treatment of breast cancer.

Anthracycline-based adjuvant regimens have become the standard of care for early-stage breast cancer in the United Kingdom. The regimen of doxorubicin and cyclophosphamide (AC) is one of a number of available choices, with widespread use in patients with an indication for chemotherapy, but a low to moderate risk of recurrence. The combination of doxorubicin and cyclophosphamide was first tested by the National Surgical Adjuvant Breast and Bowel Project as a simple alternative regimen to replace cyclophosphamide, methotrexate and 5-fluorouracil (CMF), which had been established as an effective adjuvant treatment (Fisher *et al*, 1990). In 1998, a meta-analysis of 11 randomised trials was published by the Early Breast Cancer Trialists' Collaborative Group, which explored polychemotherapy in early breast cancer. From this, it was apparent that anthracycline-containing combinations conferred a statistically significant and clinically relevant advantage in survival and recurrence compared with CMF (Early Breast Cancer Trialists' Collaborative, 1998).

A number of factors are known to influence the response to AC chemotherapy, including tumour stage, grade, number of lymph nodes involved, oestrogen receptor (ER), progesterone (PR) and ERBB2 expression (Clarke *et al*, 2008). However, possible pharmacological receptor influences on the response to adjuvant therapy have not been widely considered. In particular, there have been few investigations of the possible influence of variations in the genes encoding transporters and drug metabolising enzymes relevant for the two drugs. Doxorubicin is subject to transport by the ABCB1 transporter (Fairchild *et al*, 1987; Bradley *et al*, 1988; Leith *et al*, 1999) and by the solute transporter SLC22A16 (Okabe *et al*, 2005; Lal *et al*, 2007). Cyclophosphamide is a prodrug activated by a number of different cytochrome P450 enzymes, including CYP2B6 (Chang *et al*, 1993), CYP2C9, CYP2C19 (Chang *et al*, 1997) and CYP3A5 (Roy *et al*, 1999). Each of these enzymes and transporter genes is known to exhibit a degree of genetic variation, characterised by single nucleotide polymorphisms (SNPs) (Ingelman-Sundberg *et al*, 1999; Ariyoshi *et al*, 2001; Kim *et al*, 2001; Lal *et al*, 2007). These SNPs are present at significant frequencies in a European population and their influence on the pharmacology of a number of different agents has been characterised (Demorais *et al*, 1994; Aithal *et al*, 1999; Haas *et al*, 2004; Kimchi-Sarfaty *et al*, 2007).

The aim of this study was to examine the possible influence of SNPs on the tolerance, side effects and overall clinical outcome of AC chemotherapy in patients with breast cancer.

## Materials and methods

### Study design

A total of 230 patients were recruited to the study from 12th March 2002 until closure on 31st December 2007. All of the participants were recruited from Medical Oncology outpatient clinics within the Newcastle upon Tyne Hospitals NHS Foundation Trust and had been treated with a combination of doxorubicin and cyclophosphamide. This regimen comprises 60 mg m^−2^ doxorubicin and 600 mg m^−2^ cyclophosphamide administered intravenously on day 1 of each 21-day cycle, and repeated for a total of four or six cycles. All patients gave written informed consent and the study was given ethical approval by the Newcastle and North Tyneside Research Ethics Committee I.

A 10 ml blood sample from each patient was collected into an EDTA-containing storage tube and whole blood samples were stored at −20°C before DNA extraction. Laboratory genotyping analysis was performed on all samples for SNPs in the ABCB1, SLC22A16, CYP2B6, CYP2C9, CYP2C19 and CYP3A5 genes (details in Table 1). Clinical data were collected from patient notes and from the Trust laboratory and Patient Administration Service databases. Time to progression (TTP) and overall survival (OS) data were collected at Medical Oncology outpatient clinics, where patients attended 3-monthly from time of treatment to 2 years follow-up, 6-monthly from 2 to 5 years and annually for up to 10 years.

### Genotyping

DNA was purified from whole blood samples using a QIAmp Maxi Blood kit (Qiagen, Crawley, UK). DNA yields were estimated spectro photometrically using a Nanodrop ND-1000 (Nanodrop Technologies, Wilmington, DE, USA). Genotyping for the CYP2C9^*^2, CYP2B6^*^5, CYP2C19 and CYP3A5 SNPs was performed by the commercially available Taqman Assays on Demand method and genotyping of the SLC22A16 gene was performed by a custom-designed Taqman assay as per the manufacturer's instructions (Applied Biosystems, Warrington, UK). The CYP2B6^*^2, ^*^3 and ^*^4 SNPs were determined using a previously described RFLP method (Lang *et al*, 2001). The ABCB1, CYP2B6^*^8 and ^*^9, and CYP2C9^*^3 SNP genotyping were performed using pyrosequencing as per the manufacturer's instructions (Biotage, Uppsala, Sweden). PCR conditions for all the reactions together with primer and probe sequences and restriction enzymes used for the custom-designed assays are given in Supplementary Table 1.

### Statisitcs

A multivariate Cox proportional hazards model, applying a forward likelihood ratio, was used to assess any influence on TTP or OS. Linkage disequilibrium was explored using a *χ*^2^ analysis. Pearson's *χ*^2^ test was used to investigate the influence of SNPs on each AC chemotherapy tolerance end point (using 2 × 2 table), unless a group contained five individuals or less, when Fisher's exact test was used. To standardise these data, both TTP and OS are taken as commencing when the patient had their first AC dose, regardless of when they were recruited to the study. Kaplan–Meier charts and Cox regression were used to analyse and demonstrate the influence of SNPs on TTP and OS.

## Results

### Patient demographics

A multivariate Cox proportional hazards model, applying a forward likelihood ratio, was used to rule out any influence on TTP or OS from the following factors: age, ethnicity (Caucasian, South Asian or East Asian), histological tumour type (ductal non-specific type, lobular or other), tumour size (<20.0 mm, 20.0–49.9 mm or 50.0+ mm), tumour grade (I, II or III), unifocal or multifocal disease, number of involved axillary lymph nodes (none, 1–3 or 4+), ER status (positive or negative), PR status (positive or negative), ERBB2 status (positive or negative). ERBB2 status was only determined for 47 of the study participants as routine testing for new breast cancer presentations was introduced only in late 2005, after the date of presentation for most of our patients.

Table 2 illustrates the distribution of clinical and pathological factors within the patient population. At a median study follow-up of 63 months (range 1–107 months) 89% and 94% patients remain disease free and alive, respectively. Tumour pathology demographics were representative for patients receiving AC chemotherapy in this era.

Of the 173 (75%) of study patients with ER-positive disease, 161 received tamoxifen for a median of 46 months. Some patients received more than one type of hormonal modulation, because of a switch strategy, poor drug tolerance or extended adjuvant treatment. A total of 106 patients were treated with aromatase inhibitors (anastrazole, letrozole or exemestane) for a median of 19 months. There was no association between either tamoxifen or AI therapy and any of the SNPs as tested by *χ*^2^ analysis.

### Genotype frequencies

The distributions of the genotypes and allelotypes are shown in Table 3. All frequencies were in Hardy–Weinberg equilibrium and were consistent with those seen in previously published Caucasian populations. Linkage disequilibrium (Supplementary Table 2) was explored using Pearson's correlation co-efficient or a Fisher's exact *χ*^2^ analysis. The previously reported LD among the ABCB1 SNPs was clearly seen, and was strongest between exon the 12 and 21 SNPs. Similarly, strong LD occurred for three of the SLC22A16 SNPs, with the A146G and T132C SNPs being in 100% LD. In contrast, the T1226C SNP was actually in negative association with the A146G and T132C alleles. Strong LD was also observed for the two SNPs in CYP2B6 that contribute to the CYP2B6^*^6 allele, ^*^4 (A785G) and ^*^9 (G516T). There was a negative association between the CYP2B6^*^5 SNP and the CYP2B6^*^6 SNPs.

### Genotype and toxicity indicators

One aim of this study was to correlate genotypes in ABCB1, SLC22A16, CYP2B6, CYP2C9, CYP2C19 and CYP3A5 with tolerance of AC chemotherapy, including delivered dose intensity and toxicity. Data were gathered retrospectively from the clinical and chemotherapy records of 229 of the patients for the specific end points of ‘requirement for dose delay’, ‘requirement for dose reduction’ and ‘inability to complete planned course’. In terms of dose delays, 21% of patients experienced at least one course of AC chemotherapy where the timing of the cycle was delayed. Variant carriers, compared with homozygous wild type, of the first three linked SNPs in the SLC22A16 gene showed a statistically significant lower incidence of AC dose delay (25% *vs* 13%, *P*=0.031, 25% *vs* 13%, *P*=0.031 and 23% *vs* 8%, *P*=0.036 for A146G, T312C and T755C SNPs, respectively). Conversely, a significantly greater incidence of dose delay during AC treatment was seen in variant carriers of SLC22A16 T1226C (16% *vs* 28%, *P*=0.021), CYP2B6^*^2 (19% *vs* 55%, *P*=0.013) and CYP2B6^*^5 (18% *vs* 29%, *P*=0.053). Only 11 of the patients studied required a dose reduction and only 14 failed to complete their prescribed course. A lower incidence of dose reduction was associated with the CYP2B6^*^9 allele (8% *vs* 2%, *P*=0.020). There was no apparent influence of genotype on failure to complete course. Leucopenia and neutropenia were the most common reasons for dose delay. Carriers of the SLC22A16 T1226C minor allele also exhibited a higher incidence of leucopenia after the first cycle of therapy, compared with individuals who were wild-type homozygotes, (21% *vs* 6%, *P*=0.004). There was no association between genotype and leucopenia for any of the other SNPs studied.

### Univariate analysis of genotype and survival

The end points used for clinical outcome measures in this study were TTP and OS. The most recent clinical data showed that 89% of study participants remain disease free after AC chemotherapy.

**Figure 1:**
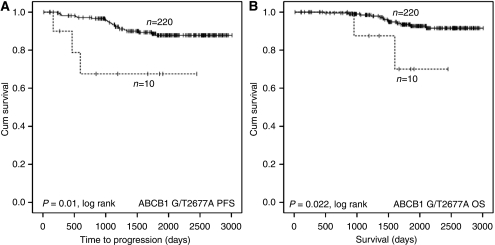
Kaplan–Meier plots illustrating impact of MDR1 genotype on progression-free survival (**A**) and OS (**B**) of breast cancer patients treated with adjuvant AC therapy with intention to cure. Curves are categorised according to MDR1 2677A allele carrier (dashed line) *vs* G2677 or 2677T allele carriers (solid line). Equality of survival distribution was tested by log rank.

Carriers of the 2677A allele (*n*=10/230) for ABCB1 demonstrated significantly shorter TTP and OS in the study population, with hazard ratios (HR) of 4.3 (CI 1.3–14.5, *P*=0.018) and 4.8 (CI 1.1–21.6, *P*=0.039), respectively, when compared to wild-type homozygotes pooled with carriers of the 2677T allele (Figure 1). There was no difference in TTP or OS associated with the other ABCB1 SNPs tested, nor the four SLC22A SNPs (Supplementary Table 3).

There was a trend towards shorter TTP and OS associated with rare allele homozygosity for the CYP2C19^*^2 SNP (Supplementary Table 3). Similarly, there was a trend towards shorter TTP associated with the CYP2B6^*^8 SNP (HR 6.772, CI 0.914–50.160, *n*=2/230, *P*=0.06) and patients heterozygous for this SNP had a significantly shorter OS compared with patients who were homozygous wild type (HR 11.906, CI 1.554–91.222, *P*=0.017). However, the relevance of these data is uncertain, given the low number of rare allele homozygotes for both SNPs (*n*=3/230 and 2/230, respectively).

**Figure 2:**
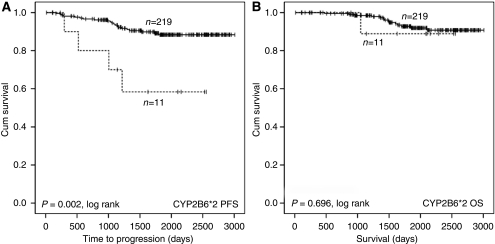
Kaplan–Meier illustrating impact of CYP2B6^*^2 genotype on progression-free survival (**A**) and OS (**B**) of breast cancer patients treated with adjuvant AC therapy with intention to cure. Curves are categorised by heterozygotes (dashed line) *vs* wild-type homozygotes (solid line). Equality of survival distribution was tested by log rank.

**Figure 3:**
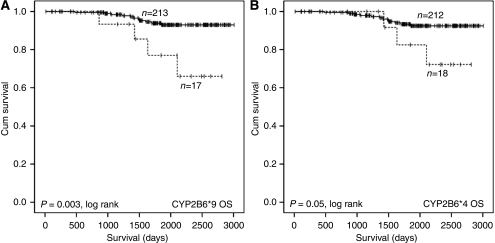
Kaplan–Meier plots illustrating impact of CYP2B6^*^4 and ^*^9 genotype on OS of breast cancer patients treated with adjuvant AC therapy with intention to cure. Curves are categorised by rare allele homozygotes for CYP2B6^*^9 (**A**) and CYP2B6^*^4 (**B**) (dashed lines) *vs* wild-type allele carriers (solid lines). Equality of survival distribution was tested by log rank.

SNPs within the CYP2B6 gene had the biggest impact on outcome in the cohort. The CYP2B6^*^2 SNP was associated with a shorter TTP (HR 4.646, CI 1.593–12.553, *n*=11/230, *P*=0.005), but with no discernable effect on OS (Figure 2A and B). The two highly linked CYP2B6 SNPs (^*^9 and ^*^4) were also associated with a poorer outcome (Figure 3A, B; Supplementary Table 3). In contrast, and consistent with the greater incidence of dose delay, there was a trend towards longer TTP in carriers of the ^*^5 allele (HR 0.325, CI 0.097–1.087, *n*=65/320, *P*=0.068; Supplementary Table 3).

The CYP2C9^*^2 and ^*^3 alleles and the CYP3A5^*^3 SNP had no effect on TTP or OS (Supplementary Table 3).

### Multivariate analysis of genotype and survival

The SNPs were subjected to multivariate Cox regression analysis using a forward stepwise likelihood ratio. The statistical significance of the association of the ABCB1 2677A allele with shorter TTP and OS was retained, as was the association of the CYP2B6^*^8 allele with shorter OS and the CYP2B6^*^2 allele with shorter TTP.

## Discussion

The combination of doxorubicin and cyclophosphamide is widely used as adjuvant treatment for breast cancer. As AC, these two drugs are also important components of the FAC and ACMF regimens. Patients selected for adjuvant treatment typically have relatively early-stage disease and have a good probability of a prolonged disease-free period. However, it is imperative to apply the appropriate regimen and dosing schedule to ensure that all patients benefit from the most effective treatment, while minimising the risk of toxicity. The recent introduction of widespread testing for ERBB2, and prescription of trastuzumab for those patients positive for this prognostic marker (Cobleigh *et al*, 1999), has highlighted the importance of treatment individualisation.

The aim of this study was to investigate the impact of pharmacogenetic factors on the tolerability of AC chemotherapy and on treatment outcome, as measured by TTP and OS. As the median follow-up in this patient group was only 62 months, there were relatively few deaths recorded at the end of the study, reducing the power of our study regarding this end point. However, the most important outcome, as regards adjuvant treatment, is TTP, as after disease recurrence, patients will usually go on to receive other treatments, which may influence OS data.

A number of genetic variants were investigated in the breast cancer patients studied. The target genes were selected on the basis of their known importance for the metabolism and transport of either doxorubicin or cyclophosphamide. For doxorubicin, the genes most likely to influence the systemic pharmacology of this drug are the transporters ABCB1 (Mechetner *et al*, 1998) and SLC22A16 (Okabe *et al*, 2005). Recent papers have investigated the importance of variations in genes that code for the carbonyl reductase enzymes, which convert doxorubicin to doxorubicinol (Lakhman *et al*, 2005). However, these investigations have been far from conclusive and relate mainly to cardiotoxicity, which was not an end point in our study.

For cyclophosphamide, the main interest is in those enzymes that are responsible for metabolic activation to form 4-hydroxycyclophosphamide. These include CYP2B6, CYP2C9, CYP2C19, CYP3A4 and CYP3A5. Of these, the impact of CYP3A4 on the activation of cyclophosphamide appears to be minimal (Chang *et al*, 1993; Roy *et al*, 1999), and despite being highly polymorphic, the identified coding SNPs occur at a low frequency in northern European populations similar to the population in this study (Eiselt *et al*, 2001). CYP2B6 has been reported to be the major enzyme involved in the activation of cyclophosphamide (Chang *et al*, 1993). This is indirectly supported by our study, as variant alleles of CYP2B6 had the greatest impact on disease outcome, with CYP2B6^*^2, ^*^8, ^*^4 and ^*^9 alleles being associated with poor prognosis. CYP2B6^*^5 was associated with a greater incidence of dose delay and may be associated with longer progression-free survival, both of which may be indicative of a higher rate of cyclophosphamide activation. However, though CYP2B6^*^2 was also associated with dose delay, paradoxically it was associated with shorter TTP, and this contradiction is currently unexplained.

Interpretation of these data in the context of the literature is made complex by the inconsistent reports on the impact of CYP2B6 SNPs on protein expression, enzymatic activity and cyclophosphamide pharmacokinetics. CYP2B6 variants have been associated with both decreased (Lang *et al*, 2001), (Jinno *et al*, 2003) and unchanged (Xie *et al*, 2003) protein expression compared with the wild-type gene. Increased specific activity associated with the variants has also been observed (Lang *et al*, 2001; Jinno *et al*, 2003). Similarly, though there are reports that CYP2B6 variants confer differences in the pharmacokinetics of cyclophosphamide (Xie *et al*, 2006), other studies have failed to show a statistically significant impact (Timm *et al*, 2005; Nakajima *et al*, 2007; Ekhart *et al*, 2008). Pharmacogenetic investigations into the impact of CYP2B6 SNPs on the pharmacokinetics of other drugs have shown fast and slow metaboliser phenotypes. For example, the CYP2B6^*^9 variant has been associated with a decreased clearance of the anti-retroviral reverse transcriptase inhibitor efavirenz (Haas *et al*, 2004; Ramachandran *et al*, 2009). Conversely, in a cohort of patients receiving thiotepa as therapy for a range of solid tumours, CYP2B6^*^5 variant carriers had a lower exposure to the CYP2B6 substrates, thiotepa and tepa, indicating a fast metaboliser phenotype (Ekhart *et al*, 2009). Given the inconsistencies in the literature and the low frequency of some of the CYP2B6 SNPs investigated in this study, these results should be treated as preliminary and require corroboration in an independent cohort.

The CYP2C19^*^2 SNP results in an aberrant splice variant that generates an alternative reading frame and a premature stop codon (Demorais *et al*, 1994). The rare allele has been associated with a lower elimination rate constant for cyclophosphamide than the wild-type allele (Timm *et al*, 2005) and a lower incidence of cyclophosphamide-induced premature ovarian failure (Takada *et al*, 2004). Other investigations have not observed a statistically significant effect on cyclophosphamide pharmacokinetics associated with the CYP2C19^*^2 SNP (Xie *et al*, 2006; Ekhart *et al*, 2008).Variants of the other CYP enzymes investigated in this study had no impact on outcome or toxicity in this cohort of breast cancer patients.

With regards to the transporters ABCB1 and SLC22A16, ABCB1 G2677T/A (‘variant A’ only) was associated with both a shorter TTP and OS, but had no impact on dose intensity. In contrast, the SLC22A16 T1226C was associated with a greater incidence of dose delay and leucopenia, but had no impact on survival. In a previous report investigating the effect of SLC22A16 genotype on doxorubicin pharmacology, only a minor effect of increased exposure to doxorubicin associated with SLC22A16 A146G rare allele homozygosity was observed, and the decreased incidence of dose delay associated with the same allele observed in this study is not consistent with an higher systemic exposure (Lal *et al*, 2007).

SLC22A16 expression in cancer cells is associated with an increased sensitivity to the cytotoxic effects of doxorubicin (Okabe *et al*, 2005). Although the functional significance of the T1226C variant form has not yet been characterised, it may be that patients with the variant genotype are able to take up more doxorubicin into both normal and tumour cells. This would be consistent with the higher incidence of dose delay in the patients carrying the variant allele. Although homozygous variants are present in less than 10% of the population, the total proportion of carriers of the variant allele is close to 40% and the effect seems to be maintained in the heterozygotes.

The other SNP found to have a significant effect on TTP was the ABCB1 G2677T/A. This non-synonymous variant results in substitution of either serine or threonine for alanine. This SNP has been widely studied, but the functional significance has not consistently been demonstrated (Lal *et al*, 2007; Nordgard *et al*, 2007). The A allele is relatively rare (3% of Caucasian population, only nine patients in this study), but the more common T allele has been reported to result in no functional change in ABCB1 activity (van den Heuvel-Eibrink *et al*, 2001) and this is reflected in the lack of effect of the T allele on treatment outcome.

Overall, a number of SNPs appeared to influence the tolerance and effectiveness of AC chemotherapy in this group of breast cancer patients, however, no formal correction for multiple testing was made and any findings should be viewed as preliminary. Further studies would be needed to validate these findings and to substantiate possible mechanisms.

## Figures and Tables

**Table 1:** SNPs investigated in AC pharmacogenetics study

**Gene**	**Ref SNP ID**	**Genotype**	**Mutation**
*ABCB1*			
Exon 12	1128503	C1236T	Synonymous
Exon 21	2032582	G2677T/A	Ala893Ser/Thr
Exon 26	1045642	C3435T	Synonymous
			
*SLC22A16*			
Exon 2	714368	A146G	His49Arg
Exon 2	6907567	T312C	Synonymous
Exon 4	723685	T755C	Val252Ala
Exon 5	12210538	T1226C	Met409Thr
			
*CYP2B6*			
^*^2	8192709	C64T	Arg22Cys
^*^8	12721655	A415G	Lys139Glu
^*^9	3745274	G516T	Gln172His
^*^3	45482602	C777A	Ser259Arg
^*^4	2279343	A785G	Lys262Arg
^*^5	3211371	C1459T	Arg487Cys
			
*CYP2C9*			
^*^2	1799853	C430T	Arg144Cys
^*^3	1057910	A1075C	Ile359Leu
			
*CYP3A5*			
^*^3	776746	A6986G	Splice variant
			
*CYP2C19*			
^*^2	4244285	G681A	Splice variant

Abbreviation: AC=doxorubicin and cyclophosphamide.

**Table 2:** Pharmacogenetic AC study patient demographics

**Demographic**	***n***	**Parameters**	**Frequency (%)**
Follow-up	230	63 months (median)	
Age range	230	57 years (median)	
			
No. progressed	230	25	
No. died	230	15	
			
Ethinicity	230	European	97
		South Asian	2
		East Asian	<1
			
Histology	229	Ductal	83
		Lobular	7
		Mixed	7
		Other	3
			
Focality	229	Unifocal	86
		Multifocal	14
			
Tumour grade	227	I	8
		II	43
		III	49
			
Tumour size	229	<20 mm	44
		20–49.9 mm	51
		⩾50 mm	5
			
Involved nodes	230	0	63
		1–3	35
		⩾4	3
			
ER status	230	Positive	75
		Negative	25
			
PR status	219	Positive	64
		Negative	36
			
ERBB2 status	47	Positive	55
		Negative	43
		Mixed	2

Abbreviations: AC=doxorubicin and cyclophosphamide; ER=oestrogen receptor; PR=progesterone receptor.

**Table 3:** Frequency results for AC study genotype and allelotype

	**Genotype frequency, *n* (%)**			**Allele frequency, *n* (%)**	
	**wt/wt**	**wt/v**	**v/v**	**wt**	**v**
ABCB1 ex12 (C1236T)	66 (29)	104 (45)	60 (26)	236 (51)	224 (49)
ABCB1 ex21 (G2677T/A)	61 (27)	wt/vT=98 (43)	vT/vT=61 (27)	224 (49)	VT=225 (49)
		wt/vA=4 (2)	vA/vA=1 (0.4)		vA=11 (2)
			vT/vA=5 (2)		
ABCB1 ex26 (C3435T)	41 (18)	112 (49)	77 (34)	194 (42)	266 (58)
SLC22A16 ex2 (A146G)	147 (64)	71 (31)	12 (5)	365 (79)	95 (21)
SLC22A16 ex2 (T312C)	147 (64)	71 (31)	12 (5)	365 (79)	95 (21)
SLC22A16 ex4 (T755C)	193 (84)	36 (16)	1 (0.4)	422 (92)	38 (8)
SLC22A16 ex5 (T1226C)	130 (57)	84 (37)	16 (7)	344 (75)	116 (25)
CYP2B6^*^2 (C64T)	219 (95)	11 (5)	0 (0)	449 (98)	11 (2)
CYP2B6^*^8 (A415G)	228 (99)	2 (1)	0 (0)	458 (99.6)	2 (0.4)
CYP2B6^*^9 (G516T)	110 (48)	103 (45)	17 (7)	323 (70)	137 (30)
CYP2B6^*^3 (C777A)	230 (100)	0 (0)	0 (0)	460 (100)	0 (0)
CYP2B6^*^4 (A785G)	125 (54)	87 (38)	18 (8)	337 (73)	123 (27)
CYP2B6^*^5 (C1459T)	165 (72)	60 (26)	5 (2)	390 (85)	70 (15)
CYP2C9^*^2 (C430T)	165 (72)	58 (25)	7 (3)	388 (84)	72 (16)
CYP2C9^*^3 (A1075C)	197 (86)	33 (14)	0 (0)	427 (93)	33 (227)
CYP3A5^*^3 (A6986G)	1 (0.4)	32 (14)	197 (86)	34 (7)	426 (93)
CYP2C19^*^2 (G681A)	163 (71)	64 (28)	3 (1)	390 (85)	70 (15)

Abbreviations: AC=doxorubicin and cyclophosphamide; wt=wild-type alllele; v=variant allele.

## References

[bib1] Aithal GP, Day CP, Kesteven PJL, Daly AK (1999) Association of polymorphisms in the cytochrome P450 CYP2C9 with warfarin dose requirement and risk of bleeding complications. Lancet 353: 717–719 10073515 10.1016/S0140-6736(98)04474-2

[bib2] Ariyoshi N, Miyazaki M, Toide K, Sawamura Y, Kamataki T (2001) A single nucleotide polymorphism of CYP2B6 found in Japanese enhances catalytic activity by autoactivation. Biochem Biophys Res Commun 281: 1256–1260 11243870 10.1006/bbrc.2001.4524

[bib3] Bradley G, Juranka PF, Ling V (1988) Mechanism of multidrug resistance. Biochim Biophys Acta 948: 87–128 2899442 10.1016/0304-419x(88)90006-6

[bib4] Chang TKH, Weber GF, Crespi CL, Waxman DJ (1993) Differential activation of cyclophosphamide and ifosphamide by cytochromes P-450 2B and 3A in human liver microsomes. Cancer Res 53: 5629–5637 8242617

[bib5] Chang TKH, Yu L, Goldstein JA, Waxman DJ (1997) Identification of the polymorphically expressed CYP2C19 and the wild-type CYP2C9-ILE359 allele as low-K-m catalysts of cyclophosphamide and ifosfamide activation. Pharmacogenetics 7: 211–221 9241661 10.1097/00008571-199706000-00006

[bib6] Clarke M, Coates AS, Darby SC, Davies C, Gelber RD, Godwin J, Goldhirsch A, Gray R, Peto R, Pritchard KI, Wood WC (2008) Adjuvant chemotherapy in oestrogen-receptor-poor breast cancer: patient-level meta-analysis of randomised trials. Lancet 371: 29–40 18177773 10.1016/S0140-6736(08)60069-0

[bib7] Cobleigh MA, Vogel CL, Tripathy D, Robert NJ, Scholl S, Fehrenbacher L, Wolter JM, Paton V, Shak S, Lieberman G, Slamon DJ (1999) Multinational study of the efficacy and safety of humanized anti-HER2 monoclonal antibody in women who have HER2-overexpressing metastatic breast cancer that has progressed after chemotherapy for metastatic disease. J Clin Oncol 17: 2639–2648 10561337 10.1200/JCO.1999.17.9.2639

[bib8] Demorais SMF, Wilkinson GR, Blaisdell J, Nakamura K, Meyer UA, Goldstein JA (1994) The major genetic-defect responsible for the polymorphism of s-mephenytoin metabolism in humans. J Biol Chem 269: 15419–15422 8195181

[bib9] Early Breast Cancer Trialists' Collaborative G (1998) Polychemotherapy for early breast cancer: an overview of the randomised trials. Lancet 352: 930–942 9752815

[bib10] Eiselt R, Domanski TL, Zibat A, Mueller R, Presecan-Siedel E, Hustert E, Zanger UM, Brockmoller J, Klenk HP, Meyer UA, Khan KK, He YA, Halpert JR, Wojnowski L (2001) Identification and functional characterization of eight CYP3A4 protein variants. Pharmacogenetics 11: 447–458 11470997 10.1097/00008571-200107000-00008

[bib11] Ekhart C, Doodeman VD, Rodenhuis S, Smits PH, Beijnen JH, Huitema AD (2008) Influence of polymorphisms of drug metabolizing enzymes (CYP2B6, CYP2C9, CYP2C19, CYP3A4, CYP3A5, GSTA1, GSTP1, ALDH1A1 and ALDH3A1) on the pharmacokinetics of cyclophosphamide and 4-hydroxycyclophosphamide. Pharmacogenet Genomics 18: 515–523 18496131 10.1097/FPC.0b013e3282fc9766

[bib12] Ekhart C, Doodeman VD, Rodenhuis S, Smits PH, Beijnen JH, Huitema AD (2009) Polymorphisms of drug-metabolizing enzymes (GST, CYP2B6 and CYP3A) affect the pharmacokinetics of thiotepa and tepa. Br J Clin Pharmacol 67: 50–60 19076156 10.1111/j.1365-2125.2008.03321.x PMC2668084

[bib13] Fairchild CR, Ivy SP, Kao-Shan C-S, Whang-Peng J, Rosen N, Israel MA, Melera PW, Cowan KH, Goldsmith ME (1987) Isolation of amplified and overexpressed DNA sequences from adriamycin-resistant human breast cancer cells. Cancer Res 47: 5141–5148 2441861

[bib14] Fisher B, Brown AM, Dimitrov NV, Poisson R, Redmond C, Margolese RG, Bowman D, Wolmark N, Wickerham DL, Kardinal CG, Shibata H, Paterson AHG, Sutherland CM, Robert NJ, Ager PJ, Levy L, Wolter J, Wozniak T, Fisher ER, Deutsch M (1990) 2 months of doxorubicin-cyclophosphamide with and without interval reinduction therapy compared with 6 months of cyclophosphamide, methotrexate, and fluorouracil in positive- node breast-cancer patients with tamoxifen-nonresponsive tumors – results from the national surgical adjuvant breast and bowel project B-15. J Clin Oncol 8: 1483–1496 2202791 10.1200/JCO.1990.8.9.1483

[bib15] Haas DW, Ribaudo HJ, Kim RB, Tierney C, Wilkinson GR, Gulick RM, Clifford DB, Hulgan T, Marzolini C, Acosta EP (2004) Pharmacogenetics of efavirenz and central nervous system side effects: an Adult AIDS Clinical Trials Group study. AIDS 18: 2391–2400 15622315

[bib16] Ingelman-Sundberg M, Oscarson M, McLellan RA (1999) Polymorphic human cytochrome P450 enzymes: an opportunity for individualized drug treatment. Trends Pharmacol Sci 20: 342–349 10431214 10.1016/s0165-6147(99)01363-2

[bib17] Jinno H, Tanaka-Kagawa T, Ohno A, Makino Y, Matsushima E, Hanioka N, Ando M (2003) Functional characterization of cytochrome P4502B6 allelic variants. Drug Metab Dispos 31: 398–403 12642465 10.1124/dmd.31.4.398

[bib18] Kim RB, Leake BF, Choo EF, Dresser GK, Kubba SV, Schwarz UI, Taylor A, Xie HG, McKinsey J, Zhou S, Lan LB, Schuetz JD, Schuetz EG, Wilkinson GR (2001) Identification of functionally variant MDR1 alleles among European Americans and African Americans. Clin Pharmacol Ther 70: 189–199 11503014 10.1067/mcp.2001.117412

[bib19] Kimchi-Sarfaty C, Oh JM, Kim IW, Sauna ZE, Calcagno AM, Ambudkar SV, Gottesman MM (2007) A ‘silent’ polymorphism in the MDR1 gene changes substrate specificity. Science 315: 525–528 17185560 10.1126/science.1135308

[bib20] Lakhman SS, Ghosh D, Blanco JG (2005) Functional significance of a natural allelic variant of human carbonyl reductase 3 (CBR3). Drug Metab Dispos 33: 254–257 15537833 10.1124/dmd.104.002006

[bib21] Lal S, Wong ZW, Jada SR, Xiang XQ, Shu XC, Ang PCS, Figg WD, Lee EJD, Chowbay B (2007) Novel SLC22A16 polymorphisms and influence on doxorubicin pharmacokinetics in Asian breast cancer patients. Pharmacogenomics 8: 567–575 17559346 10.2217/14622416.8.6.567

[bib22] Lang T, Klein K, Fischer J, Nussler AK, Neuhaus P, Hofmann U, Eichelbaum M, Schwab M, Zanger UM (2001) Extensive genetic polymorphism in the human CYP2B6 gene with impact on expression and function in human liver. Pharmacogenetics 11: 399–415 11470993 10.1097/00008571-200107000-00004

[bib23] Leith CP, Kopecky KJ, Chen IM, Eijdems L, Slovak ML, McConnell TS, Head DR, Weick J, Grever MR, Appelbaum FR, Willman CL (1999) Frequency and clinical significance of the expression of the multidrug resistance proteins MDR1/P-glycoprotein, MRP1, and LRP in acute myeloid leukemia. A Southwest Oncology Group Study. Blood 94: 1086–1099 10419902

[bib24] Mechetner E, Kyshtoobayeva A, Zonis S, Kim H, Stroup R, Garcia R, Parker RJ, Fruehauf JP (1998) Levels of multidrug resistance (MDR1) P-glycoprotein expression by human breast cancer correlate with *in vitro* resistance to taxol and doxorubicin. Clin Cancer Res 4: 389–398 9516927

[bib25] Nakajima M, Komagata S, Fujiki Y, Kanada Y, Ebi H, Itoh K, Mukai H, Yokoi T, Minami H (2007) Genetic polymorphisms of CYP2B6 affect the pharmacokinetics/pharmacodynamics of cyclophosphamide in Japanese cancer patients. Pharmacogenet Genomics 17: 431–445 17502835 10.1097/FPC.0b013e328045c4fb

[bib26] Nordgard SH, Ritchie MD, Jensrud SD, Motsinger AA, Alnaes GIG, Lernmon G, Berg M, Gelsler S, Moore JH, Lonning PE, Borresen-Dale AL, Kristensen VN (2007) ABCB1 and GST polymorphisms associated with TP53 status in breast cancer. Pharmacogenet Genomics 17: 127–136 17301692 10.1097/FPC.0b013e328011abaa

[bib27] Okabe M, Unno M, Harigae H, Kaku M, Okitsu Y, Sasaki T, Mizoi T, Shiiba K, Takanaga H, Terasaki T, Matsuno S, Sasaki I, Ito S, Abe T (2005) Characterization of the organic cation transporter SLC22A16: A doxorubicin importer. Biochem Biophys Res Commun 333: 754–762 15963465 10.1016/j.bbrc.2005.05.174

[bib28] Ramachandran G, Ramesh K, Hemanth Kumar AK, Jagan I, Vasantha M, Padmapriyadarsini C, Narendran G, Rajasekaran S, Swaminathan S (2009) Association of high T allele frequency of CYP2B6 G516T polymorphism among ethnic south Indian HIV-infected patients with elevated plasma efavirenz and nevirapine. J Antimicrob Chemother 63: 841–843 19218571 10.1093/jac/dkp033

[bib29] Roy P, Yu LJ, Crespi CL, Waxman DJ (1999) Development of a substrate-activity based approach to identify the major human liver P-450 catalysts of cyclophosphamide and ifosfamide activation based on cDNA-expressed activities and liver microsomal P-450 profiles. Drug Metab Dispos 27: 655–666 10348794

[bib30] Takada K, Arefayene M, Desta Z, Yarboro CH, Boumpas DT, Balow JE, Flockhart DA, Illei GG (2004) Cytochrome P450 pharmacogenetics as a predictor of toxicity and clinical response to pulse cyclophosphamide in lupus nephritis. Arthritis Rheum 50: 2202–2210 15248218 10.1002/art.20338

[bib31] Timm R, Kaiser R, Lotsch J, Heider U, Sezer O, Weisz K, Montemurro M, Roots I, Cascorbi I (2005) Association of cyclophosphamide pharmacokinetics to polymorphic cytochrome P4502C19. Pharmacogenomics J 5: 365–373 16116487 10.1038/sj.tpj.6500330

[bib32] van den Heuvel-Eibrink MM, van Schaik RHN, van der Heiden I, Sonneveld P, Pieters R, Wiemer EAC (2001) MDR-1 gene polymorphisms G2677T and C3435T do not correlate with P-glycoprotein expression and function in acute myeloid leukemia (AML). Blood 98: 130211520775

[bib33] Xie H, Griskevicius L, Stahle L, Hassan Z, Yasar U, Rane A, Broberg U, Kimby E, Hassan M (2006) Pharmacogenetics of cyclophosphamide in patients with hematological malignancies. Eur J Pharm Sci 27: 54–61 16183265 10.1016/j.ejps.2005.08.008

[bib34] Xie HJ, Yasar U, Lundgren S, Griskevicius L, Terelius Y, Hassan M, Rane A (2003) Role of polymorphic human CYP2B6 in cyclophosphamide bioactivation. Pharmacogenomics J 3: 53–61 12629583 10.1038/sj.tpj.6500157

